# Imaging the efficacy and side effects of CAR T-cell therapy in children and young adults

**DOI:** 10.1186/s40644-026-01004-0

**Published:** 2026-02-12

**Authors:** Iryna Vasyliv, Zahra Shokri Varniab, Shashi B. Singh, Claire Johns, Sneha Ramakrishna, Kara L. Davis, Heike E. Daldrup-Link

**Affiliations:** 1 Division of Pediatric Radiology, Department of Radiology, Stanford University School of Medicine, Stanford, CA USA; 2 Department of Pediatrics, Bass Center for Childhood Cancer and Blood Disorders, Center for Cancer Cell Therapy, Stanford University, Stanford, CA USA; 3 Department of Pediatrics–Hematology/Oncology, Lucile Packard Children’s Hospital, Stanford University, Stanford, CA USA

**Keywords:** CAR T-cell therapy, Pediatric oncology, Imaging biomarkers, Cytokine release syndrome, ICANS, Treatment response, Immunotherapy

## Abstract

CAR T-cell therapy is a transformative immunotherapy for pediatric patients with relapsed or refractory malignancies. Imaging plays a crucial role in evaluating treatment efficacy and detecting complications. This review provides time-specific guidance for radiologists on the imaging findings before and after CAR T-cell infusion and highlights how imaging supports clinical decision-making across the treatment course. Pre-treatment imaging at baseline focuses on ruling out active infection and quantifying residual tumor burden. In addition, quantitative imaging biomarkers can predict tumor response and toxicity risk. Between day 0–28 after CAR T-cell infusion, imaging is primarily performed to detect side effects, such as cytokine release syndrome (CRS) and immune effector cell-associated neurotoxicity syndrome (ICANS). At day + 28, imaging provides early assessment of tumor response. Radiologists must recognize atypical immune-related response patterns at this time, including pseudoprogression and, less commonly, hyperprogression. Beyond day 28, imaging monitors for late side effects and infections, while also documenting ongoing tumor response or recurrence. Familiarity with these time-specific imaging patterns and pitfalls, coupled with knowledge of quantitative biomarkers, enables radiologists to differentiate toxicity from therapeutic effect, avoid misclassification of early immune-related changes, and optimize outcomes in pediatric CAR T-cell therapy.

## Background

Chimeric Antigen Receptor (CAR) T-cell therapy reprograms a patient’s own T lymphocytes to recognize and eliminate malignant cells, functioning as a living, adaptive drug [1]. In pediatric hematologic malignancies, CD19-directed CAR T-cell products are FDA-approved [2], while newer constructs targeting CD22 and dual antigens are under active investigation [3]. Imaging plays a crucial role in evaluating treatment efficacy, and some patients exhibit remarkably robust responses. However, the existing literature on medical imaging of CAR-T cell therapies of pediatric patients remains limited to small cohorts and case descriptions (Table 1). To address this gap, our article will summarize the existing imaging literature, which is drawn largely from adult CAR-T studies and to a lesser extent pediatric cohorts, and complement it with our own experience imaging children and young adults undergoing CAR-T therapy.

**Table 1:** Summary of published CAR-T imaging original research and case reports in pediatric and young adult patients

Author (year)	Journal	Patient age	Tumor Type	Imaging Modality	Conclusion
McGuire, J. L. et al. 2025	*Neurology*	12 (median age)	B-Cell Malignancies	Brain MRI	ICANS-related brain MRI abnormalities demonstrate unique patterns in the cerebral white matter, brainstem and thalami; their prevalence increases with ICANS clinical grade.
Tan et al. 2020	*Pediatr Blood Cancer*	Pediatric cohort (7 patients)	B-ALL	Brain MRI	Acute neuroimaging findings may be a potential imaging biomarker for peak neurotoxicity and treatment response, and it is not necessarily associated with poor outcome, as previously reported.
Kim et al. 2025	*Eur Radiol*	11–21	B-ALL	Brain MRI	Children and young adults with B-ALL can develop brain MRI abnormalities after CAR T-cell therapy, predominantly WM signal changes. These brain abnormalities did not show an association with higher CRS or ICANS grade.
Monje et al., 2025	*Nature*	4–30	H3K27M⁺ diffuse midline glioma	Brain MRI	Sequential IV, followed by ICV GD2-CART, induced tumour regressions and neurological improvements in patients with DIPG and those with sDMG.
Wang et al., 2019	*Biol. Blood Marrow Transplant*	22–67	Relapsed/refractory non-Hodgkin lymphoma	PET/CT	Data indicate that patients with higher baseline disease burden have more severe CRS, and that CAR-T cell therapy is associated with lymphoma pseudoprogression and local immune activation.
Vasyliv et al., 2025	*Scientific Reports*	4–25	B-ALL	Chest CT	In children and young adults with ALL, increased thymus size after CAR T-cell therapy was associated with younger age and improved clinical outcomes.
**Author (year)**	**Journal**	**Patient age**	**Tumor Type**	**Imaging Modality**	**Key Findings**
Ko et al. 2024	Korean J Radiol	20	Relapsed ALL	CT	Localized CRS with cervical soft-tissue edema; complete resolution after steroids.
Andrew et al. 2023	Br J Haematol	14; 12	B-ALL	Brain MRI	Persistent severe white matter abnormalities with diffusion restriction; progressive deep white matter & brainstem involvement; did not resolve for months.
Kim et al. 2025	Eur Radiol	11–21	B-ALL	Brain MRI	Children and young adults with B-ALL can develop brain MRI abnormalities after CAR T-cell therapy, predominantly WM signal changes. These brain abnormalities did not show an association with higher CRS or ICANS grade.
Huang et al., 2021	*Immunotherapy*	6-year-old boy	B-ALL	MRI	Pseudoprogression observed as asymptomatic lesion enlargement on day 16, regressing by day 30 after anti-CD19 CAR T therapy.
Cohen et al., 2022	*Eur. J. Nucl. Med. Mol. Imaging*	23-year-old female	Refractory diffuse large B-cell lymphoma	PET/CT	Immune-related pseudoprogression at day 15 (SUVmax 22.8, Deauville 5); biopsy showed inflammation; near-complete metabolic resolution by day 42.
Guo et al., 2024	*World J. Nucl. Med.*	13-year-old boy	Relapsed/refractory rhabdomyosarcoma	PET/CT	Hyperprogression at day 28 with numerous new and enlarging FDG-avid nodules (SUVmax up to 28.6) after B7H3/CD171 CAR T-cell therapy.
Wang G. et al., 2023	*Clin. Nucl. Med*	16-year-old girl	Recurrent ALL	PET/CT	Pulmonary tuberculosis 1 month post CD19 CAR T-cell therapy; FDG-avid mediastinal nodes and lung nodule mimicked relapse; PPD confirmed infection/reactivation.
Xu et al., 2020	*Pediatric Investigation*	4-year-old boy	Neuroblastoma with MYCN amplification	MRI and MIBG scintigraphy	Sustained response of occipital and sphenoid bone lesions at 18 weeks and 37.5 months post GD2-CAR T-cell therapy.
Perrone et al., 2024	*Anticancer Research*	12-year-old girl	B-ALL	PET/CT	Solitary occult breast relapse detected at day + 120 after CD19 CAR T-cell therapy.
Du & Zhang, 2020	*Journal of Cancer Research and Clinical Oncology*	8-year-old boy	Relapsed/refractory Burkitt lymphoma	PET/CT	Sequential CD19, CD22, CD20 CAR T-cell therapies: transient response then remission; complete metabolic remission post CD20 CAR T, event-free for 16 months.
Mu et al., 2023	*Frontiers in Immunology*	17-year-old male	Large cell lymphoma	PET/CT	Pseudoprogression at day + 88 after anti-CD5 CAR T; biopsy showed CAR T expansion, not relapse; resolution by day + 255.
Kawata et al., 2023	*Cancer Reports*	14-year-old girl	B-ALL	Chest CT	Bilateral ground-glass opacities (“crazy-paving” pattern) consistent with Pneumocystis jirovecii pneumonia 4 months post CAR T therapy.
Gray & Fair, 2025	*Clinical Nuclear Medicine*	23-year-old male	B-ALL→ Myeloid sarcoma	PET/CT	Development of myeloid sarcoma 1.5 months after CD19 CAR T, showing numerous FDG-avid lesions across multiple organs.
An et al., 2024	*International Journal of Hematology*	9-year-old boy	Histiocytic sarcoma (lineage-switched relapse)	MRI	Development of histiocytic sarcoma 3 months post CD19/22 CAR T-cell therapy, presenting with massive hepatomegaly and multiple enhancing, diffusion-restricting liver lesions.
Zhang et al., 2023	*Frontiers in Cellular and Infection Microbiology*	23–63	Hematologic malignancies	Chest CT	Active tuberculosis developed within 23 days − 11 months post CAR T therapy.

**Fig. 1:**
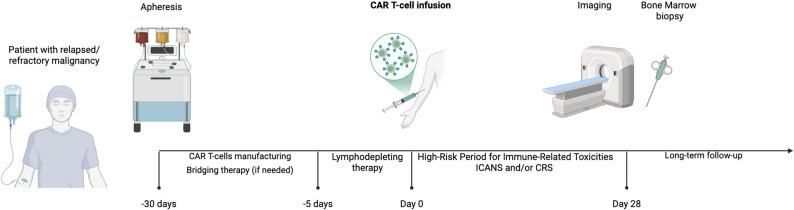
Timeline of imaging and response assessment in CAR T-cell therapy: baseline, early post-treatment monitoring (days 0–28), day 28, and long-term follow-up

Our review is organized along the clinical timeline of CAR T-cell therapy (Fig. 1), because distinct imaging findings emerge at different stages: Approximately 30–60 days before CAR T-cell infusion, patients undergo T-cell apheresis. The collected T-cells are genetically modified to express a specific CAR that target tumor-specific antigens. If necessary, bridging chemotherapy may be administered to prevent tumor progression [4]. At this stage, baseline imaging studies are conducted to exclude active infection and quantify residual tumor burden. Approximately one week before CAR T-cell infusion, patients are hospitalized to receive lymphodepleting conditioning therapy (typically from day − 5 to -2) which reduces circulating lymphocytes and enhance CAR T-cell efficacy [5]. Once in aplasia, CAR T-cells are infused with patients managed in standard hospital rooms without routine isolation, depending on institutional protocols and clinical status (day 0) [6]. Importantly, pediatric patients do not routinely require stem cell transplant level of isolation, as lymphodepleting chemotherapy and CAR T-cell infusion generally do not confer a higher infectious risk than standard chemotherapy regimens. During the next 2–3 weeks, patients are closely monitored for potential inflammatory responses or toxicities. During this time, imaging studies are primarily conducted to evaluate complications such as CRS and ICANS [7]. At 2–3 weeks post CAR T-cell infusion, patients are typically discharged. On approximately day + 28 after CAR T-cell infusion, imaging is used to determine tumor therapy response, along with assessment of bone marrow biopsy [8]. This assessment may occur in either the inpatient or outpatient setting, depending on ongoing toxicities and clinical status. Thereafter, long term follow up imaging focuses on the detection of late toxicities and monitoring tumor response. Applying a time-based framework allows for distinction of imaging findings that arise at each stage of CAR T-cell treatment.

## Imaging at baseline

**Fig. 2:**
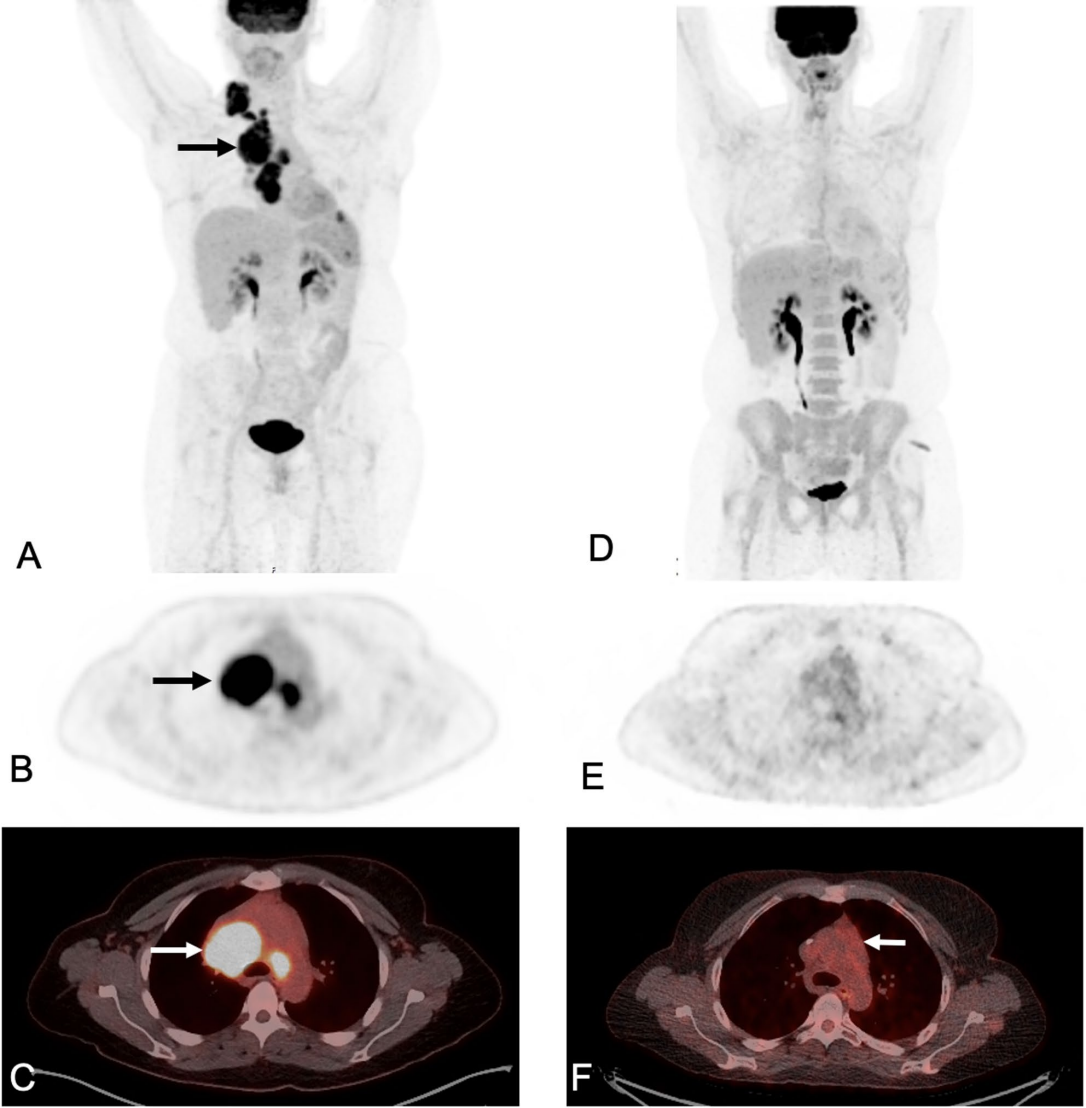
18F-FDG PET/CT of a 34-year-old female with diffuse large B-cell lymphoma who achieved a complete metabolic response after CD19/20 CAR T-cell therapy.** (A–C)** Baseline 18F-FDG PET/CT: **A**) Baseline MIP demonstrates FDG-avid thoracic lymphadenopathy (black arrow) consistent with active lymphoma. **B)** Axial PET demonstrates hypermetabolic anterior mediastinal lymph nodes activity (black arrow) **C)** Baseline fused axial 18F-FDG PET/CT confirms multiple hypermetabolic anterior mediastinal lymph nodes (white arrows). **(D–F)** Follow up 18F-FDG PET/CT at day 28 after CAR T-cell infusion **D**) Follow-up MIP at day 28 after CAR T-cell infusion shows complete metabolic response. **E**) Axial PET confirms complete metabolic response to therapy with decrease in size and FDG uptake of the previously noted thoracic lymphadenopathy. No new sites of FDG avid disease noted. **F**) Follow up fused axial 18F-FDG PET/CT confirms decrease in size and FDG uptake of multiple mediastinal lymph nodes (white arrows)

Baseline imaging serves to rule out active infection before lymphodepleting therapy and to establish the extent of residual tumor burden prior to CAR T-cell infusion. Baseline tumor burden has generally been inversely associated with CAR T-cell therapy response. Nevertheless, children and young adults can exhibit impressive responses, even when presenting with relatively high baseline tumor burden (Fig. 2).

 Predicting tumor response: Quantitative 18F-FDG PET parameters such as maximum standardized uptake value (SUVmax), metabolic tumor volume (MTV), and total lesion glycolysis (TLG) have emerged as powerful predictors of treatment efficacy. Several studies reported that baseline tumor SUVmax and Total Lesion Glycolysis (TLG) measurements can predict survival in adult patients (Table 2). Gui et al. reported that baseline SUVmax ≥ 10.9 and Total Lesion Glycolysis (TLG) ≥ 72.1 in adults with diffuse large B-cell lymphoma were associated with worse overall survival [9]. Breen et al. found that pre-leukapheresis to pre-lymphodepletion increases in extranodal MTV ≥ 25% and TLG of the largest lesion ≥ 10% identified patients with aggressive Non-Hodgkin lymphoma at higher risk of disease progression or death [10]. Similarly, Sjöholm et al. observed that baseline MTV ≤ 39.5 mL, TLG ≤ 308.9, and ΔADCmean > 0.92x10^-6^ mm2/s predicted significantly longer PFS and OS in patients with large B-cell lymphoma [11]. Galli et al. showed that MTV > 48.4 cm³ and increased LDH strongly predicted poor progression-free survival, whereas low MTV (< 48.4 cm³) and normal LDH had indicated better outcomes [12]. Likewise, Hong et al. reported that adult NHL patients with baseline tumor SUVmean ≥ 4.36, MTV ≥ 26.37 cm³, and TLG ≥ 78.61 (SUV·cm³) had significantly lower one-year survival [13]. Alderuccio et al. reported that MTV with a cutoff point of 96 mL at baseline predicted event-free survival of adult patients with diffuse large B-cell lymphoma [14]. To our knowledge, there are no systematic reports to date, whether these thresholds also apply to children.

**Table 2:** Publications reporting quantitative metrics on baseline 18F-FDG PET scans that were associated with progression-free survival (PFS) or overall survival (OS) in patients with lymphoma after CAR T-cell therapy

Author (year)	Journal	Patient age	Lymphoma type	PET parameters	Prognostic association
Gui et al., 2019	Eur J Nucl Med Mol Imaging	29–74	DLBCL	SUVmax ≥ 10.9; TLG ≥ 72.1	Worse overall survival
Breen et al., 2023	Blood Cancer J	26–76	NHL	MTV ↑ ≥25%; TLG ↑ ≥10%	Higher risk of progression or death
Sjöholm et al., 2022	Cancer Imaging	14–76	Large B-cell lymphoma	MTV ≤ 39.5 mL; TLG ≤ 308.9	Longer PFS and OS
Galli et al., 2025	Hematol Oncol	28–75	B-cell lymphoma	MTV > 48.4 cm³	Poor PFS
Hong et al., 2021	Front Oncol	25–71	NHL	SUVmean ≥ 4.36; MTV ≥ 26.37 cm³; TLG ≥ 78.61	Lower 1-year survival
Alderuccio et al., 2024	Clin Cancer Res	62.2 (mean)	DLBCL	MTV ≥ 96 mL	Predicted event-free survival

Abbreviations: 18F-FDG, fluorine-18 fluorodeoxyglucose; PET, positron emission tomography; PFS, progression-free survival; OS, overall survival; SUV, standardized uptake value; SUVmax, maximum SUV; SUVmean, mean SUV; MTV, metabolic tumor volume; TMTV, total metabolic tumor volume (sum of MTV across lesions); TLG, total lesion Glycolysis (MTV multiplied by SUVmean)

**Fig. 3:**
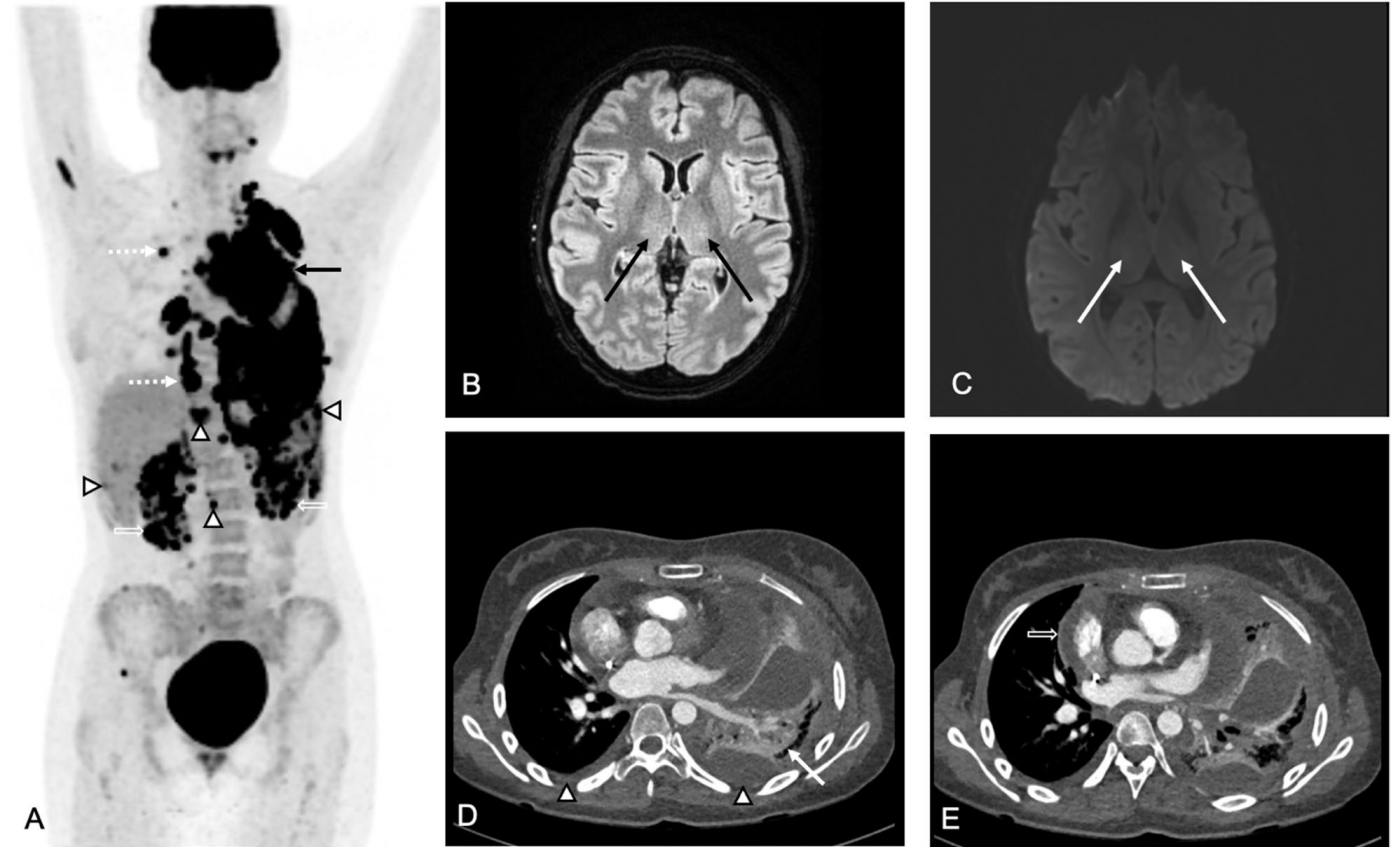
19-year-old female with diffuse large B-cell lymphoma and high baseline metabolic tumor burden who developed grade 2 cytokine release syndrome (CRS) and grade 3 immune effector cell–associated neurotoxicity syndrome (ICANS) after CAR T-cell therapy.** A)** 18F-FDG PET MIP at baseline demonstrates a bulky hypermetabolic anterior mediastinal mass (black arrow), extensive 18F-FDG-avid lymphadenopathy above and below the diaphragm (dashed arrow arrowheads) as well as multiple hypermetabolic lesions in the bilateral kidneys (open arrows), spleen (open arrowhead), liver (open arrowhead) and bone marrow (open arrowheads). **B)** Axial FLAIR MR image of the brain at day 6 after the CAR T-cell injection shows mild bilateral thalamic T2-hyperintensity (black arrows). **C)** Diffusion-weighted MR image of the brain at day 6 reveals subtle restricted diffusion in the bilateral thalami (white arrows). **D)** Axial contrast-enhanced CT image demonstrates a small right pleural effusion and a moderate left pleural effusion (arrow heads), consistent with CRS-related capillary leak syndrome. The adjacent lung demonstrates normal contrast enhancement, consistent with atelectasis rather than pneumonia (white arrow). **E)** Axial contrast-enhanced CT image through the level of the heart demonstrates associated pericardial effusion (open arrow)

**Fig. 4:**
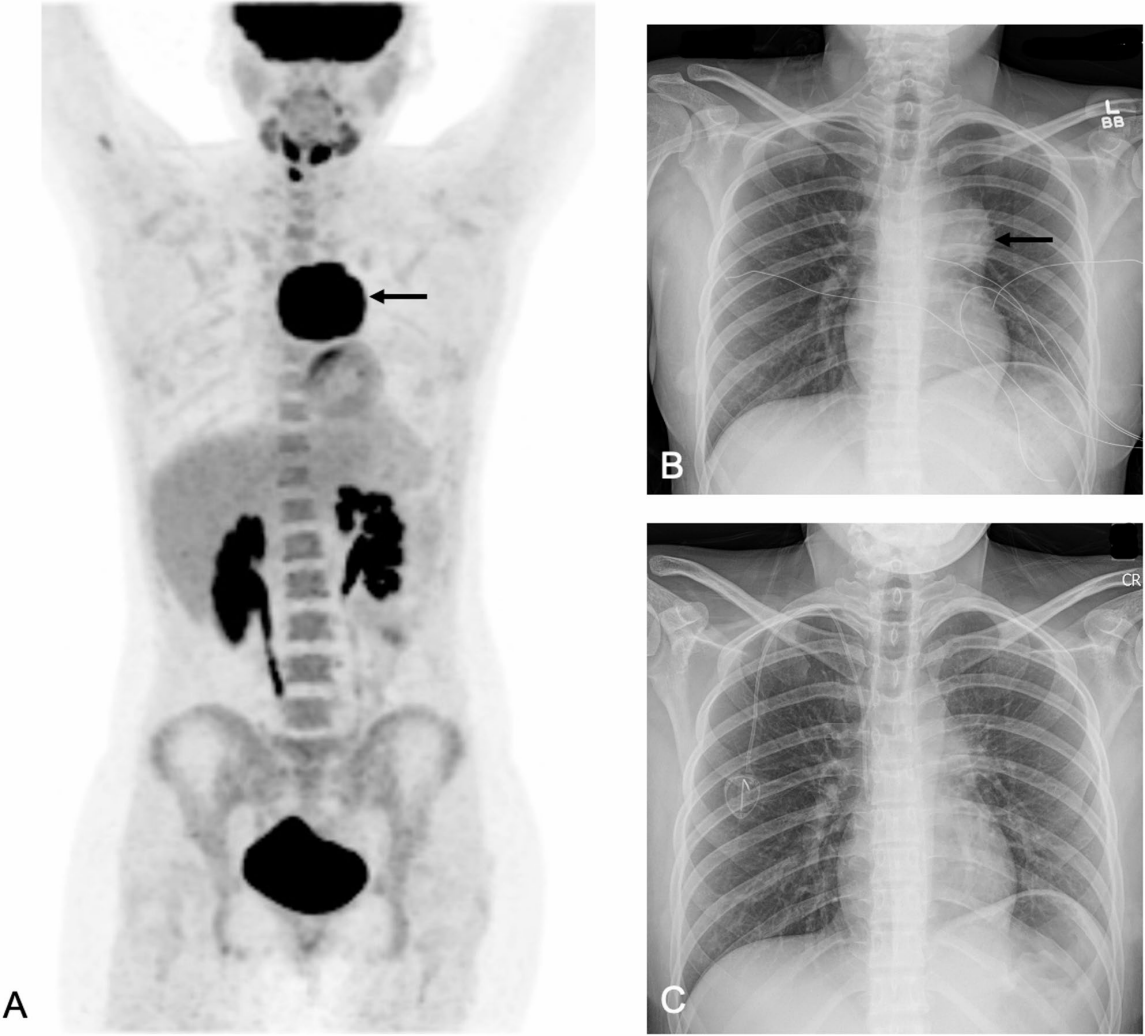
19-year-old female with mediastinal large B cell lymphoma and relatively low metabolic tumor burden at baseline who did not develop CRS or ICANS after CAR T-cell therapy.** A)** Baseline PET MIP shows anterior mediastinal mass (black arrow). No other lesions were noted. **B)** Baseline Chest radiograph demonstrates mild widening of the mediastinum, consistent with the mediastinal mass (black arrow). **C)** Chest radiograph on day 9 after the CAR T-cell injection demonstrates mild widening of the mediastinum. No new pleural effusion or pulmonary infiltrates, consistent with an uncomplicated post–CAR T-cell clinical course

 Predicting toxicities: Baseline imaging metrics can also predict the risk of immune-mediated toxicities in normal organs. Multiple studies in adult patients reported that high baseline tumor burden was associated with increased CAR T-cell activation, cytokine release, systemic inflammation and downstream complications: Gui et al. reported that baseline tumor SUVmax correlated significantly with the severity of CRS in adult patients with diffuse large B-cell lymphoma (rₛ = 0.806, *P* < 0.001) [9]. Hong et al. reported that a baseline tumor SUVmean ≥ 4.36, MTV ≥ 26.37 cm³, and TLG ≥ 78.61 (SUVxcm³) was significantly associated with severe CRS in adults with non-Hodgkin lymphoma [13]. Breen et al. observed that pre-lymphdepletion total MTV of 221 cc median was associated with grade ≥ 3 ICANS in patients with aggressive Non-Hodgkin lymphoma [10]. Our experience confirmed these patterns in children and young adults (Figs. 3 and 4).

### Imaging on day 0–28

Following lymphodepleting chemotherapy, patients enter a phase of aplasia that increases their susceptibility to infections. In addition, CAR T-cell infusion can trigger a spectrum of immune-mediated toxicities. To mitigate these risks, patients are typically hospitalized and may be admitted to an isolation unit, allowing for intensive monitoring and early intervention. Fever is common during the early post-infusion period and overlaps with neutropenia, leading to empiric broad-spectrum antibiotic therapy. As a result, classical imaging manifestations of infection may be absent or subtle, and routine infection-directed imaging is not commonly performed unless there is clinical deterioration or lack of response to antimicrobial therapy. However, subclinical infections may already be present during the first 28 days but remain undetected due to limited imaging and blunted inflammatory responses. This is supported by the observation that up to one quarter of patients show signs of infection at day 28 [15]. Imaging findings of infections in days 0–28 are often nonspecific, and clinicians should maintain a high index of suspicion for occult infection. Medical imaging studies during the early post-infusion phase are mostly done to investigate inflammatory complications related to immune activation, such as CRS and ICANS.

### Cytokine release syndrome (CRS)

CRS is a common complication of CAR T-cell therapy, affecting most patients. While usually mild, 10% to 30% of patients develop severe forms (grade 3–4) [16]. CRS typically presents within the first two weeks post-infusion, although delayed onset can occasionally occur. Clinical manifestations include fever, generalized edema, hypotension, hypoxia, and multi-organ dysfunction. The severity of CRS is clinically monitored by elevated cytokines levels, particularly IL-6, which is central to its pathogenesis and can be effectively mitigated with IL-6 inhibitor therapy [17].

**Fig. 5:**
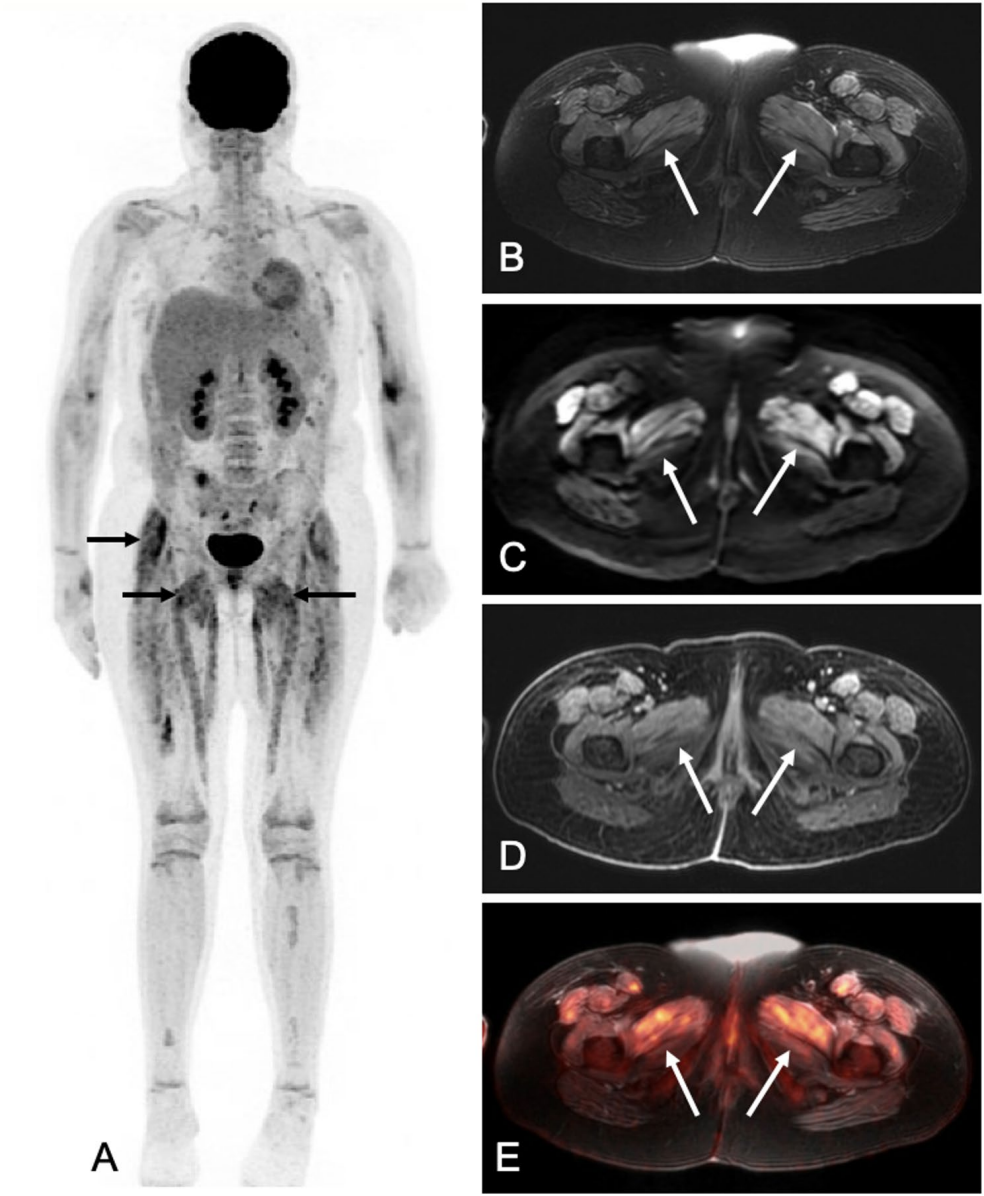
CRS-induced myositis in a 13-year-old girl with relapsed acute lymphoblastic leukemia, noted on 18F-FDG PET/MR at day 28 after CAR T-cell infusion. **A**) 18F-FDG PET MIP demonstrates marked 18F-FDG radiotracer uptake in skeletal muscle throughout the body, including the bilateral legs (arrows), consistent with myositis. ​**B**) Axial T2-weighted MRI at the level of the pelvis shows bilateral diffuse T2-hyperintense edema of muscles of the anterior thigh, including the adductor muscles.​ **C**) Axial diffusion-weighted MRI at the level of the pelvis shows mildly restricted diffusion in the affected muscles with ADCmean of 980x 10^−6^ mm2/s. **D**) Axial contrast-enhanced T1-weighted MRI shows mild contrast enhancement of affected muscles. **E**) Integrated 18F-FDG PET/MR demonstrates contrast enhancement and 18F-FDG radiotracer uptake in the affected muscles, consistent with myositis

**Fig. 6:**
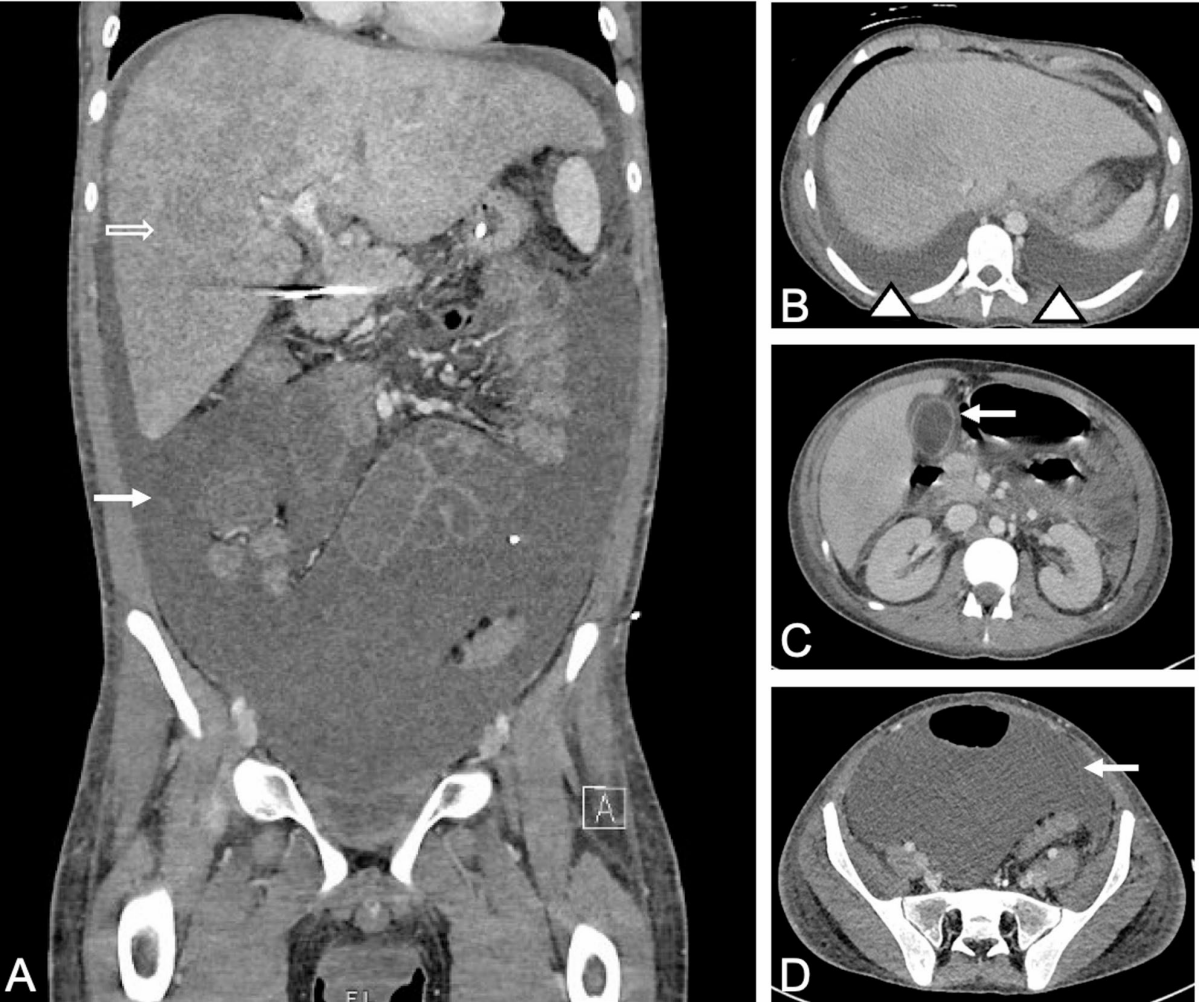
CRS in a 16-year-old young man with relapsed B-cell acute lymphoblastic leukemia, noted on a contrast-enhanced CT scan at 12 days following CAR T-cell therapy.** A)** Coronal contrast-enhanced CT demonstrates massive ascites (white arrow) and marked liver edema (open arrow). **B)** Axial scan through the lower chest shows bilateral pleural effusions (arrowheads). **C)** Axial CT through the upper abdomen demonstrates pericholecystic fluid (white arrow) and blunted enhancement of the kidneys, consistent with renal edema. **D)** Axial scan through the lower abdomen demonstrates extensive ascites (arrow). Findings are consistent with CRS-induced capillary leakage

Imaging findings of CRS include signs of capillary leak and third spacing of fluid, such as edema of subcutaneous soft tissues and visceral organs, ascitis, pleural effusions, pericardial effusions, and noncardiogenic pulmonary edema [18]. CRS can occasionally present in a localized form. Ko et al. reported a case of a 20-year-old female with relapsed ALL and CRS after CD19-targeted CAR T-cell infusion who developed localized edema of cervical soft tissues, diagnosed on CT. The finding resolved completely after steroid therapy [19]. In our experience, children with CRS–induced capillary leak often develop fluid accumulation and tissue edema across multiple compartments. Chest radiographs and chest CT may show pleural and pericardial effusions, pulmonary edema, and soft tissue edema. Abdominal US, CT or MRI can demonstrate ascitis, visceral organ edema, including hepatic and renal swelling, intestinal wall thickening and diffuse subcutaneous edema. CRS can also manifest as myositis, characterized by T2-hyperintense muscle signal on MRI and increased 18F-FDG uptake on PET imaging (Fig. 5). The degree of third spacing and involvement of affected organs increases with increasing CRS grade (Fig. 6).

**Fig. 7:**
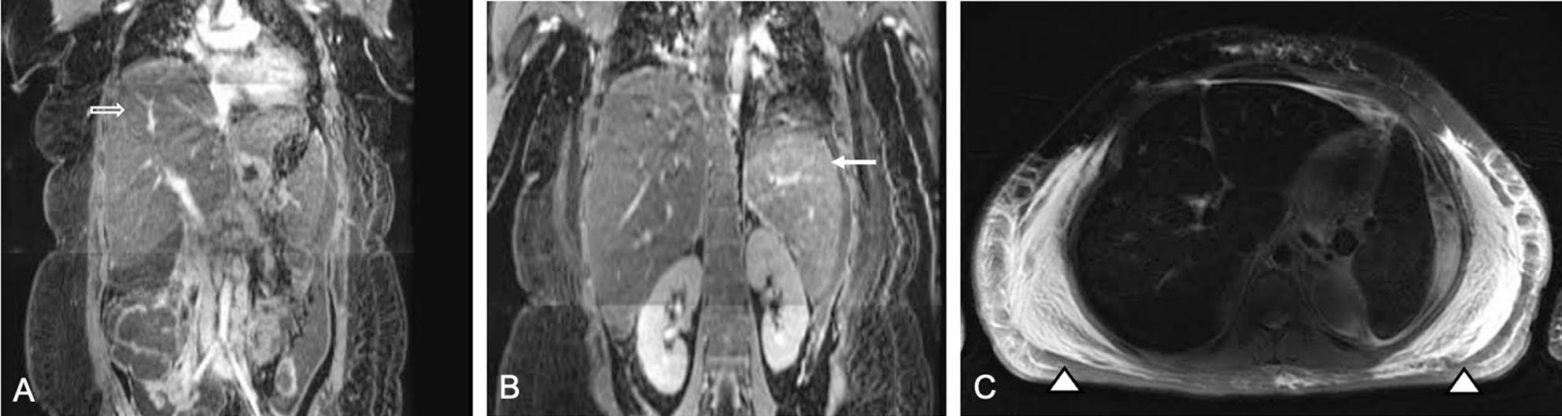
Contrast-enhanced MRI scans of a 18-year-old woman with relapsed B-cell acute lymphoblastic leukemia, who developed hemophagocytic lymphohistiocytosis at day 42 after CAR T-cell therapy. **A**) Coronal contrast-enhanced T1-weighted MRI shows marked edema and swelling of the liver (open arrow, 23.8 cm in craniocaudal dimension). **B**) Coronal contrast-enhanced T1-weighted MRI shows marked enlargement of spleen (arrow, 16.2 cm in craniocaudal dimension). **C**) Axial T2-weighted MRI through the level of the liver and spleen shows marked subcutaneous soft tissue edema (arrow heads) and diffuse low T2-signal of the liver and spleen due to severe iron overload

 CAR T-cell-induced Hemophagocytic Lymphohistiocytosis (HLH) is a severe hyper-inflammatory syndrome which is characterized by excessive activation of the immune system, leading to widespread inflammation and multi-organ dysfunction. Medical imaging findings are similar to those of high-grade CRS and may include splenomegaly, lymphadenopathy, pleural effusions and ascites (Fig. 7). Martín-Rojas et al. reported a 34-year-old man who developed HLH following CD19-directed CAR T-cell therapy. PET/CT at baseline showed extensive tumor burden. PET/CT on day + 25 showed a paradoxical metabolic response of lymphoma lesions despite worsening clinical symptoms. Unfortunately, the report did not include PET images, so it remains unclear whether the scan also showed evidence of organ toxicity. Bone marrow biopsy demonstrated hemophagocytosis without lymphoma infiltration. Despite clinically confirmed CAR T-cell expansion, tumor response and corticosteroid, siltuximab, and anakinra therapy to treat HLH, the patient developed multiorgan failure and died on day + 36 [20]. This case highlights that radiologists must interpret medical imaging findings in the broader clinical context, as complete metabolic responses do not exclude life-threatening systemic immune complications.

### Immune effector cell–associated neurotoxicity syndrome (ICANS)

ICANS is a neuroinflammatory complication of CAR T-cell therapy. In a study with 45 adult patients with large B-cell lymphoma, Holtzman et al. reported ICANs in 25 (56%) patients, of whom 18 (72%) had severe grade 3–4 symptoms [21]. By contrast Kim et al. reported a lower incidence in a small series of 16 children with B-cell ALL, with only three patients developing grade 1–2 ICANS and none developing grade 3–4 ICANS [22].

Clinical signs include headaches, disorientation, visual disturbances, aphasia, tremor, ataxia, fine motor impairment, and seizures [23]. Elevated cytokines such as interleukin-6 (IL-6), interferon-γ (IFN-γ), IL-10, IL-15, IL-2, and TNF-α can disrupt the blood-brain barrier (BBB) and lead to cerebral edema, and neurotoxicity [24]. Treatment primarily focuses on supportive care and immunosuppression with anti-seizure prophylaxis and corticosteroids [25].

**Fig. 8:**
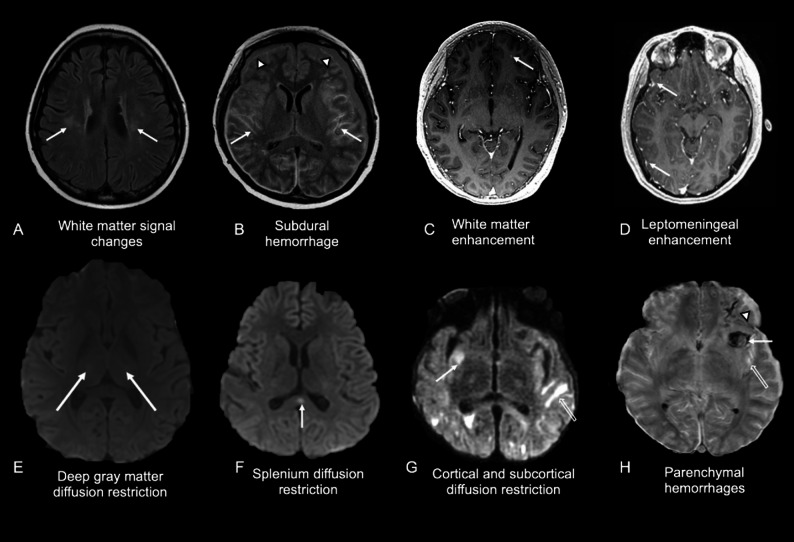
Spectrum of brain MRI findings in different patients with ICANS after CAR T-cell therapy.** A)** Axial T2 FLAIR MR image of a 17-year-old female patient at day 21 after CAR T-cell therapy shows hyperintense lesions in the bilateral cerebral white matter (arrows). **B)** Axial T2 FLAIR MR image of an 15-year-old female patient at one year after CAR T-cell therapy and 2 days after cytotoxic T-lymphocyte infusion shows combination of left temporal and inferior frontal superficial parenchymal hemorrhage and subarachnoid hemorrhage as well as superficial right temporal parenchymal hemorrhage (white arrows) and bilateral subdural collections along the cerebral convexities (arrowheads) resulting in mild mass effect on the cerebral hemispheres. **C)** Axial contrast-enhanced T1-weighted MR image of an 11-year-old female patient at day 16 after CAR T-cell therapy shows a small focal contrast-enhancing lesion in the left frontal white matter (arrow). **D)** Axial contrast-enhanced T1-weighted MR image of an 18-year-old male patient at day 4 after CAR T-cell therapy shows leptomeningeal enhancement in the right middle cranial fossa (arrows). **E**) Axial diffusion-weighted MR image of a 19-year-old female patient at day 6 after CAR T-cell therapy shows an area of symmetric restricted diffusion in the thalamus (arrows). **F**) Axial diffusion-weighted MR image of a 17-year-old female patient at day 3 after CAR T-cell therapy shows focal restricted diffusion in the splenium of the corpus callosum (arrow). **G**) Axial diffusion-weighted MR image of a 15-year-old female patient at day 5 after CAR T-cell therapy shows both areas of cortical (arrows) and subcortical (open arrows) restricted diffusion in the temporal and occipital lobes. **H**) Axial gradient echo MR image of a 15-year-old female patient at day 14 after CAR T-cell therapy shows combination of parenchymal hemorrhage in left temporal and inferior frontal lobe (white arrow), subarachnoid hemorrhage of left temporal lobe (open arrow) and left frontal subdural collection (arrowhead)

 Brain MRI abnormalities have been reported in adult patients with B-ALL treated CD19 CAR T-cell out of 53 patients 27 (51%) developed ICANS; 19 underwent brain MRI during acute symptoms, and 6 patients (32% of those imaged) demonstrated ICANS-related MRI abnormalities [26], among 864 pediatric patients with B-cell malignancies treated with CD19 and/or CD19/22-directed CAR T-cells, 343 (40%) developed ICANS, and 96 (11%) underwent acute brain MRI; of these, 36% showed ICANS-related MRI changes [27], seven pediatric patients with B-ALL treated with 19 CAR T-cell therapy showed clinical signs of neurotoxicity and four of them had ICANS-related MRI findings [28] and 13 (81%) out of 16 pediatric patients with B-ALL treated with CD19 or CD19/22 CAR T-cell therapy demonstrated MRI abnormalities, but only 3 patients had ICANS [22]. The spectrum of observed brain MRI findings in pediatric patients after CAR T-cell therapy is shown in Fig. 8. Most common findings included white matter hyperintensities on T2-weighted and fluid-attenuated inversion recovery (FLAIR) sequences [22], similar to leukoencephalopathy after methotrexate therapy [29]. No significant correlation was noted between white matter T2/FLAIR lesion burden and the clinical severity of CRS or ICANS [22]. Additional CNS findings included T2-hyperintense edema in the thalami, caudate nuclei, pons, medulla, cingulate gyri, and hippocampi in pediatric patients with B-cell malignancies [27]. Other rare findings included small areas of acute ischemic injury, and leptomeningeal enhancement [22]. Agarwal et al. described a 30-year-old woman with B-ALL who developed grade 3 ICANS at day 5 after anti-CD19 CAR T-cell therapy and symmetric bilateral cortical and subcortical T2/FLAIR hyperintensities in the parieto-occipital lobes on MRI, without contrast enhancement, consistent with posterior reversible encephalopathy syndrome (PRES) [30]. Treatment with pulse methylprednisolone and anti-cytokine therapy led to complete clinical recovery and radiologic resolution by day 69.

**Fig. 9:**
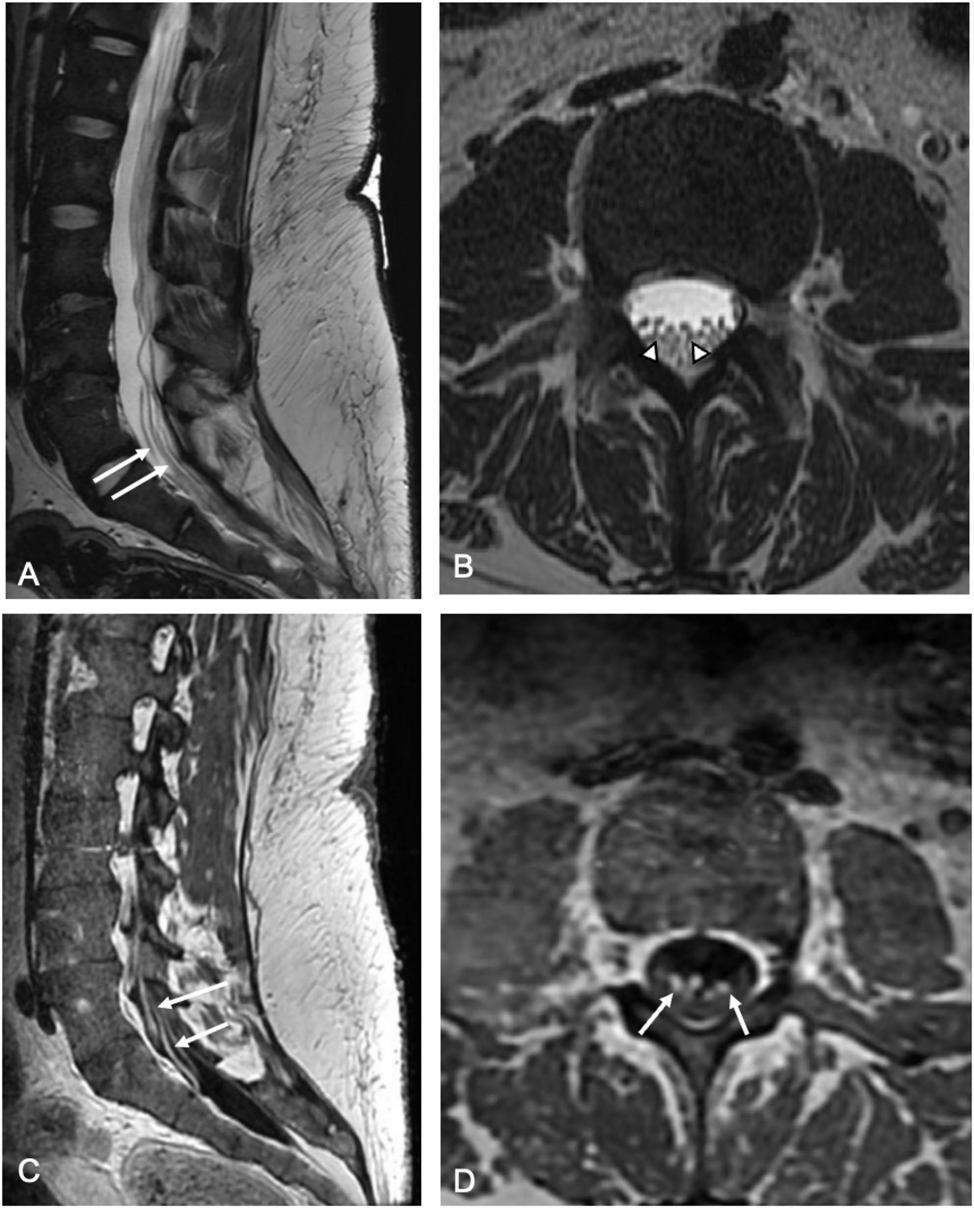
Cauda equina polyneuritis at day 13 after CAR T-cell infusion in a 25-year-old woman with acute lymphoblastic leukemia and subacutely worsening weakness of lower extremity weakness. (A) Sagittal and (B) axial T2-weighted MR images of the lumbar spine demonstrate thickening of multiple cauda equina nerve roots (white arrows and arrowheads). C) Sagittal and D) axial contrast-enhanced T1-weighted MR images demonstrate enhancement of the thickened nerve roots consistent with diffuse polyneuritis (white arrows)

Spinal cord MRI abnormalities: Several authors reported cases of spinal cord involvement after anti-CD19 CAR T-cell therapy. Khanli reported a 29-year-old woman with relapsed/refractory diffuse large B-cell lymphoma who developed high-grade ICANS and T2/FLAIR-hyperintense, non-enhancing signal abnormalities of the medulla, cervical and thoracic spinal cord which resolved after steroid therapy [31]. Similarly, Sheikh et al. described a 28-year-old woman with refractory primary mediastinal large B-cell lymphoma who developed grade 4 ICANS and MRI findings of transverse myelitis, with increasing T2/FLAIR-hyperintense signal abnormalities and contrast enhancement of the dorsal central thoracic cord between day + 27 to + 65. Follow-up spine MRI at day + 208 after corticosteroids showed marked improvement of MRI findings, consistent with clinical recovery [32]. Tan et al. reported diffuse leptomeningeal enhancement two patients after CD19 CAR T-cell therapy [28]. We observed a similar case in a 25-year-old woman with B-ALL who had no ICANS but presented on day 13 after CD19-directed CAR T-cell infusion with subacute worsening of upper-extremity weakness on a background of chronic lower-extremity weakness. Her spinal MRI demonstrated abnormal enhancement of multiple cervical ventral nerve roots and ventral cauda equina nerve roots. Neurological symptoms improved after treatment with intravenous immunoglobulin (Fig. 9).

Brain and spinal cord abnormalities after CAR T-cell therapy can be transient or chronic. Santomasso et al. reported complete resolution of T2/FLAIR hyperintensities in the bilateral thalami/brainstem and splenium at day’s range 4–21 days [26]. Tan reported persistence or progression of white matter signal abnormalities at 28–35 day, followed by partial resolution at 8 months [28]. Andrew et al. reported that a 12-year-old girl with multiply relapsed B-ALL developed progressive deep white-matter T2/FLAIR hyperintensities with restricted diffusion along the corticospinal tracts, brainstem, and cerebellar peduncles persisting through day + 54, while a 14-year-old boy showed persistent deep white-matter T2/FLAIR hyperintensities with restricted diffusion through day + 78, despite high-dose corticosteroids and intravenous immunoglobulin [33].

### Imaging on day 28–30

Imaging data on optimal response assessment after CAR T-cell therapy are still limited, current evidence comes from CAR T-specific studies. In a prospective study of 41 patients with relapsed and/or refractory lymphoma (large B-cell lymphoma, follicular lymphoma, mantle-cell lymphoma) treated with CD19 CAR T cells, Winkelmann et al. obtained baseline CT or PET/CT within 2 weeks before infusion and follow-up imaging at approximately day 30 and day 90 after therapy to compare four established lymphoma response criteria (Lugano, Cheson, RECIL and LYRIC). Overall response rates at day 90 were similar across criteria (63–68%), but progressive disease rates differed markedly (32% Lugano, 27% Cheson, 17% RECIL, 17% LYRIC), highlighting that both choice of response criteria and the standardized imaging time points around 1 and-3 months after CAR T-cell infusion substantially influence reported outcomes [34]. To summarize these findings, Table 3 provides an overview of commonly used lymphoma response assessment systems and their associated imaging modalities.

**Table 3:** Response assessment systems used in lymphoma and their corresponding imaging modalities

Response system	Imaging modality	Response Criteria
Lugano	PET	1: No uptake. 2: Uptake ≤ mediastinal blood pool. 3: Uptake > mediastinal blood pool but ≤ liver. 4: Uptake moderately higher than the liver. 5: Uptake markedly higher than the liver and/or new lesions. Clinical Interpretation Negative (Complete Response): Scores 1, 2, and 3. Positive (Partial/No Response): Scores 4 and 5.
RECIL	Conventional imaging or PET	Complete Response: disappearance of all target lesions, with nodes having a long axis < 10 mm. Must be combined with PET negativity (Deauville score 1–3) and no bone marrow involvement. Partial Response: at least 30% reduction in the sum of the longest diameters of target lesions, plus a Deauville score of 4 or 5 (indicating residual PET uptake) or a 30% reduction with PET negativity. Minor Response: a 10% to < 30% reduction in sum of the longest diameters. Stable Disease: a reduction in sum of the longest diameters of < 10% or an increase of ≤ 20%. Progressive Disease: an increase in sum of the longest diameters of > 20% from the lowest value (nadir), or the appearance of any new lesion.
iRECIST	Conventional imaging or PET	Immune Complete Response: disappearance of all target and non-target lesions; lymph nodes must decrease to < 10 mm. Immune Partial Response: ≥ 30% decrease in the sum of diameters of target lesions compared to baseline. Immune Stable Disease: Neither Immune Partial Response nor Immune Unconfirmed Progressive Disease criteria are met. Immune Unconfirmed Progressive Disease: ≥ 20% increase in sum of diameters (minimum 5 mm increase) compared to nadir, or appearance of new lesions. Immune Confirmed Progressive Disease: Confirmed Immune Unconfirmed Progressive Disease on a subsequent scan (4–8 weeks later), showing further increase in tumor burden, persistent new lesions, or additional new lesions.
LYRIC	PET	Indeterminate Response (IR) Definitions: IR(1): ≥ 50% increase in sum of the product of diameters of lesions within the first 12 weeks, with no clinical deterioration. IR(2): New lesions or ≥ 50% increase in existing lesion size, but total tumor burden increase is < 50%. IR(3): Increased ^18^F-FDG uptake in lesions without a corresponding increase in size or number

Several studies demonstrated that metabolic and volumetric tumor responses at day 28 can predict outcomes in patients with relapsed/refractory large B-cell lymphoma (Table 3). Farolfi et al. reported that an SUVmax ≥ 9.1 at day 28 after infusion of CD19-, CD22- or CD19-targeted CAR T-cells was significantly associated with inferior overall and progression-free survival [35]. Other authors reported thresholds of tumor SUVmax ≥ 5.69 SUVmax ≥ 7 [36], SUVmax ≥ 10 [37], and SUVmax ≥ 14 [38]. Similarly, Galtier et al. reported that patients with relapsed/refractory large B-cell lymphomas and a Deauville score of 5 at day 30 after CAR T-cell therapy had a 1-year progression free survival of ~ 8.6% versus ~ 70–80% 1-year PFS in those with Deauville 1–4 responses [39]. In this cohort Deauville 4 did not represent true residual tumor activity but rather post-treatment inflammatory uptake and therefore behaved prognostically similar to complete metabolic responders (DS1–3). Other measures associated with unfavorable outcomes were MTV > 30 cm³ [36] and MTV ≥ 60.8 mL [35] as well as TLG ≥ 23.79 [9] and TLG ≥ 97.0 [35] at day 28.

Liang et al. reported that patients with chronic lymphocytic leukemia who achieved a complete metabolic response on day 28 18F-FDG PET-CT, defined as normalization of bone marrow and lymph node uptake to levels below liver background, demonstrated markedly improved outcomes, including longer progression-free survival (median 8.9 months), prolonged duration of response (median 18.9 months), and better overall survival (median 25.0 months) compared with those without a day 28 complete response [40].

 Imaging findings of normal organs that reflect treatment response: Derlin et al. analyzed serial ¹⁸F-FDG PET/CT in ten patients with relapsed or refractory diffuse large B-cell lymphoma treated with CD19-targeting CAR T-cell therapy. They found that an early reduction in metabolic activity of the spleen (SUVmean − 42% ± 22%) between baseline and day + 30 post-infusion was significantly associated with poor outcome. Meanwhile, patients maintaining near-baseline splenic and nodal metabolism (− 7% ± 10% and + 4% ± 28%) achieved partial or complete remission at day + 90 [41]. Early post CAR T PET CT studies suggest that increased splenic FDG uptake is a nonspecific marker of systemic immune activation. In patients with relapsed or refractory classic Hodgkin lymphoma treated with anti–PD-1 immune-checkpoint inhibitors (nivolumab or pembrolizumab), Dercle et al. reported that increased splenic FDG metabolism at 3-month PET/CT was associated with treatment response: among the 16 patients studied, the 9 responders (56%) demonstrated a significant rise in spleen SUV compared with baseline, whereas non-responders did not show this metabolic increase [42]. The same heightened immune activation can also be associated with inflammatory toxicities. An increased 18F-FDG uptake in the spleen has been associated with activation of humoral immune responses [43], innate immune responses including immune effector cell-induced lymphohistiocytosis-like syndrome (IEC-HS) [44, 45], and systemic inflammation [43]. By around 3 months post-infusion, splenic uptake typically trends back toward baseline in most patients, even if they had CRS. If persistently increased splenic activity is noted at 3 months, ongoing or new triggers should be considered, such as HLH/MAS-like toxicity or prolonged immune activation, Intercurrent infection or inflammation, transfusion effects or hemolysis, splenic recurrence of malignancy. Therefore, elevated splenic activity should not be interpreted as uniformly favorable or unfavorable in isolation, but rather as a sign of immune activation that must be correlated with clinical status.

An increased 18F-FDG uptake (SUV) of the liver may indicate immune cell activation, because the liver plays a central role in clearing circulating cytokines and immune complexes. ^90^ Beck et al. reported that an increase in Liver SUVmean between baseline and day 28 scans of + 11.3 ± 15.4% (4.9–22.7%) was associated with durable remission in adult patients with diffuse large B cell lymphoma after CD19-targeted CAR-T cell therapy [46].

**Fig. 10:**
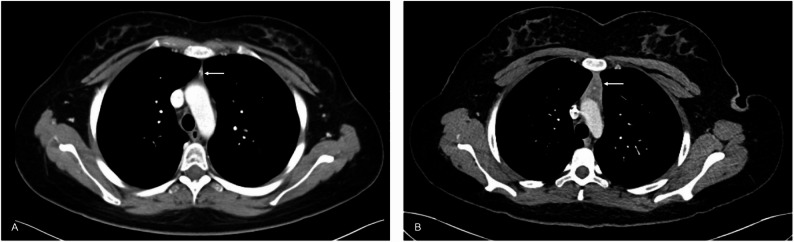
Thymus enlargement after CAR T-cell therapy in a 16-year-old female patient with B-ALL.** A)** Baseline axial contrast-enhanced chest CT shows a small amount of thymic tissue in the anterior mediastinum (arrow). **B)** Axial CT image at day 52 after CAR T-cell therapy demonstrates benign thymic enlargement with smooth contours and mild, homogenous contrast enhancement (arrow). The patient demonstrated a robust tumor response with relapse-free survival for four years

In children, thymic enlargement after CAR T-cell therapy can indicate favorable response. An active thymus can produce thymic emigrants (RTEs), i.e. T helper cells that support CAR T proliferation and anti-tumor activity. Thymic enlargement on imaging studies at 1–3 months after CAR T therapy, together with higher CD4⁺ recent thymic emigrants, was a strong predictor of event-free survival (Fig. 10) [47].

**Fig. 11:**
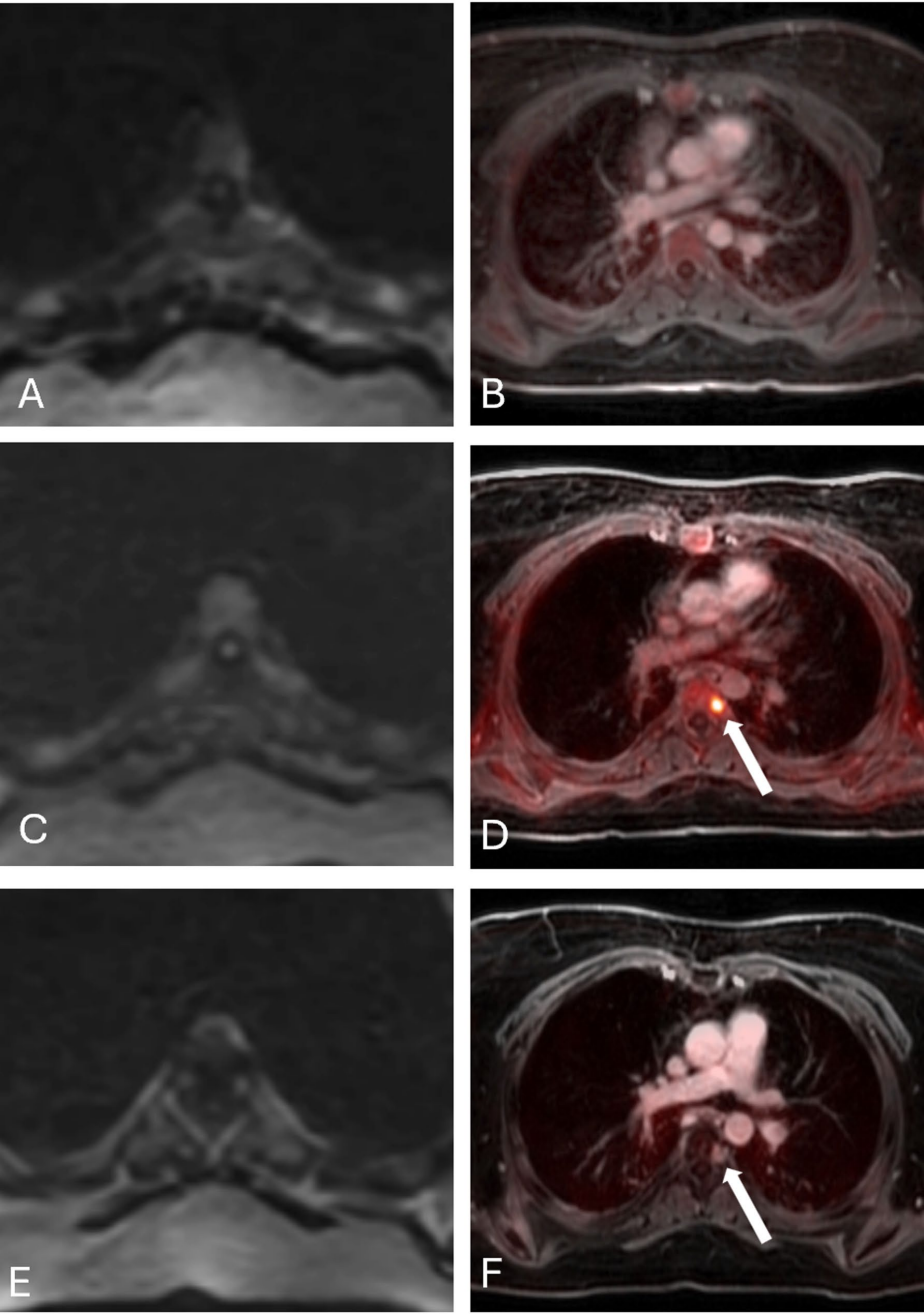
Pseudoprogression after CAR T-cell infusion in a 26-year-old female with osteosarcoma. **A**) Baseline axial diffusion weighted MRI (DWI) of the mid thoracic spine demonstrates a small lesion with slightly restricted diffusion in the left T5 vertebral body (arrow). **B**) Baseline fused PET/MR demonstrates normal 18F-FDG metabolism of the bone marrow. **C**) Day 28 axial DWI shows restricted diffusion of the entire T5 vertebral body, consistent with increased cell density in the bone marrow, likely a treatment effect. **D**) Day 28 fused 18F-FDG PET/MR demonstrates a small new 18F-FDG avid lesion in the left T5 vertebral body (arrow). **E**) DWI at 3 months after CAR T-cell infusion demonstrates normalization of marrow MRI signal. **F**) 3-month fused PET/MR shows resolution of 18F-FDG uptake on the PET scan and increased MRI contrast enhancement of the lesion, consistent with therapy response (arrow)

 Pseudoprogression describes a temporary increase in tumor size or metabolic activity because of immune cell infiltration and activation after immunotherapy. Pseudoprogression can complicate the interpretation of early post-treatment scans, making it challenging to differentiate treatment-related changes from actual tumor progression. In patients with solid tumors undergoing CAR T-cell therapy, observed apparent new metabolic activity in small bone marrow lesions at day 28, which resolved on subsequent follow-up scans (Fig. 11). A meta-analysis of eight original research studies in lymphoma patients receiving immune checkpoint inhibitors reported pseudoprogression in 10% of children and adult patients [48]. Danylesko et al. reported signs of pseudoprogression in 14 of 56 (25%) adult patients with aggressive B-cell non-Hodgkin lymphoma treated with CAR T-cell therapy [49]. Wang et al. reported pseudoprogression in 3 out of 19 adult patients (16%) with relapsed or refractory non-Hodgkin lymphoma treated with CD19-directed CAR T-cell therapy, developing within 4–6 days post-infusion and resolving spontaneously within 1–2 weeks. Patients with pseudoprogression demonstrated a median metabolic tumor volume of 267.2 cm³ and a median total lesion glycolysis of 2698.7, compared with 49.3 cm³ and 394.2 in patients without pseudoprogression [50]. Thus far, findings of pseudoprogression in pediatric patients is limited to individual case reports. For example, Huang et al. reported pseudoprogression in a 6-year-old boy with relapsed B-ALL and extramedullary bone and soft tissue involvement treated with anti-CD19 CAR T-cell therapy. Follow-up MRI on day 16 demonstrated asymptomatic lesion enlargement, which regressed by day 30 [51]. Cohen et al. reported a 23-year-old female with refractory diffuse large B-cell lymphoma treated with CD19-targeted CAR T-cell therapy who developed cervical swelling and dyspnea [52]. Day 15 PET/CT demonstrated increased ^18^FDG uptake in cervical lymph nodes (SUV max 22.8, Deauville 5) and new ^18^FDG-avid foci, raising concern for disease progression, but biopsy revealed only inflammation. By day 42, ^18^FDG PET/CT showed near-complete metabolic resolution (SUV max 3.4, Deauville 3, ΔSUV max > 66%), confirming immune-related pseudoprogression. In our experience, pseudoprogression is frequently accompanied by systemic immune activation, such as myositis or imaging features of CRS. Although CRS can occur in both true progression and pseudoprogression, the absence of systemic inflammation argues against pseudoprogression.

 Rapid relapse and hyperprogression: Patients can rarely demonstrate tumor hyperprogression at day 28, rapidly progressive tumor growth. Hyperprogression is the paradoxical acceleration of tumor growth beyond pre-treatment kinetics occurring early after CAR T-cell therapy, without any meaningful initial response. This needs to be differentiated from rapid tumor relapse after initial response (Table 4). Guo et al. described tumor hyperprogression in a 13-year-old patient with relapsed, therapy-refractory rhabdomyosarcoma after B7H3 and CD171 targeted CAR T-cells. The patient developed numerous new and enlarging solid tumor nodules in the muscles, subcutaneous tissues, and skin of the left lower limb on the first post-treatment 18F-FDG PET scan at day 28, with tumor SUV max up to 28.6 [53] (Table 5).

**Table 4:** Publications reporting quantitative 18F-FDG PET metrics at day 28–30 post–CAR T-cell therapy that were associated with progression-free survival (PFS) or overall survival (OS) in patients with lymphoma

Author (year)	Journal	Patient age	Lymphoma type	PET parameters	Prognostic association
Farolfi et al., 2025	J. Nucl. Med.	48–65	Large B-cell lymphoma	SUVmax ≥ 9.1, MTV ≥ 60.8 mL, TLG ≥ 97.0	Inferior PFS and OS
Sesquez et al., 2022	Clin. Nucl. Med	27–78	B-cell lymphoma	SUVmax ≥ 14 of residual/recurrent lesions	Inferior OS
Zaki et al., 2023	Blood Adv.	18–84	Large B-cell lymphoma	SUVmax < 10	Complete remission
Lutfi et al., 2023	Clin. Lymphoma Myeloma Leuk.	22–75	Large B-cell lymphoma	SUVmax ≥ 7, TMV ≥ 30 cm³	Inferior outcomes
Gui et al., 2024	Eur J Nucl Med Mol Imaging	29–74	Diffuse large B-cell lymphoma	SUVmax ≥ 5.69, TLG ≥ 23.79	Shorter PFS

Abbreviations: 18F-FDG, fluorine-18 fluorodeoxyglucose; PET, positron emission tomography; PFS, progression-free survival; OS, overall survival; SUV, standardized uptake value; SUVmax, maximum SUV; SUVmean, mean SUV; MTV, metabolic tumor volume; TMTV, total metabolic tumor volume (sum of MTV across lesions); TLG, total lesion Glycolysis (MTV multiplied by SUVmean)

**Table 5:** Imaging characteristics of hyperprogression versus rapid relapse

Feature	Hyperprogression	Rapid Relapse After Response
Initial response	No or minimal	Yes, transient response
Duration of benefit	None or < 1 month	Weeks, new or enlarging lesion within < 3 months
Growth kinetics	Faster than pre-treatment	May be fast, but after initial response period
Timing	At first assessment	After documented response
Comparison	Worse than natural history	Disease returns or progresses after transient disease control

 Infection/inflammation: While the primary focus of day 28 imaging is on assessment of tumor response, imaging studies may also reveal signs of infection or inflammation. Most infections at day 28 after CAR T-cell therapy arise from prolonged neutropenia. Isolation units and prophylactic antibiotics reduce exposure risk but cannot prevent endogenous infections related to central lines, mucositis, and low white cell counts. Hill et al. found signs of infections in 30 of 133 patients (23%) treated with CD19 CAR T cells at day 28, including pneumonia, C. difficile colitis, perirectal/soft-tissue abscesses, bacterial sinusitis, pyelonephritis/urinary tract infection and invasive fungal infections (Aspergillus) of the lungs and sinuses [15]. Wang G et al. reported pulmonary tuberculosis in a 16-year-old girl with recurrent ALL one month after CD19-directed CAR T-cell therapy [54]. FDG-avid mediastinal lymph nodes and a pulmonary nodule were initially mistaken for tumor relapse, but a positive PPD test confirmed the diagnosis of infection, which might have been due to reactivation of a previously latent mycobacterium.

### Imaging beyond day 28: long term follow up

While some patients develop long-term responses, others develop transient response with eventual tumor recurrence or progression. Compared with adults, children often have better baseline T-cell fitness and longer persistence that can translate into more durable remissions. Monje et al. reported that one child with H3K27M+ diffuse midline glioma demonstrated sustained antitumor responses on MRI at 30 months post GD2-CAR T-cell therapy [55]. Xu et al. reported sustained response of occipital and sphenoid bone lesions in a child with neuroblastoma on MRI and MIBG scintigraphy studies at 18 weeks and 37.5 months after treatment with GD2-targeted CAR T-cells [56].

When solid tumors eventually relapse, tumor recurrences may occur in unusual and unexpected locations. Perrone et al. described a solitary occult breast relapse in a 12-year-old girl with ALL day + 120 following CD19 CAR T-cell therapy [57].

While rare, late presentations of pseudoprogression can also occur. Mu et al. described a 17-year-old male with large-cell lymphoma who presented with enlarged, hypermetabolic cervical and inguinal lymph nodes on 18F-FDG PET/CT at day + 88 after anti-CD5 CAR-T cell infusion. Biopsy demonstrated expanded CAR-T cells rather than relapsed lymphoma, and day + 255 follow-up imaging demonstrated resolution [58].

Cytopenia and immunosuppression often persist for many months or even years after CAR T-cell therapy, leading to risk for atypical infections. In children, infection risk from prolonged aplasia and hypogammaglobulinemia can be higher and more prolonged compared to adults [59, 60]. Zhang et al. reported five patients who developed active tuberculosis within ~ 192 days (23days-11months) after CAR T-cell therapy [61].

**Fig. 12:**
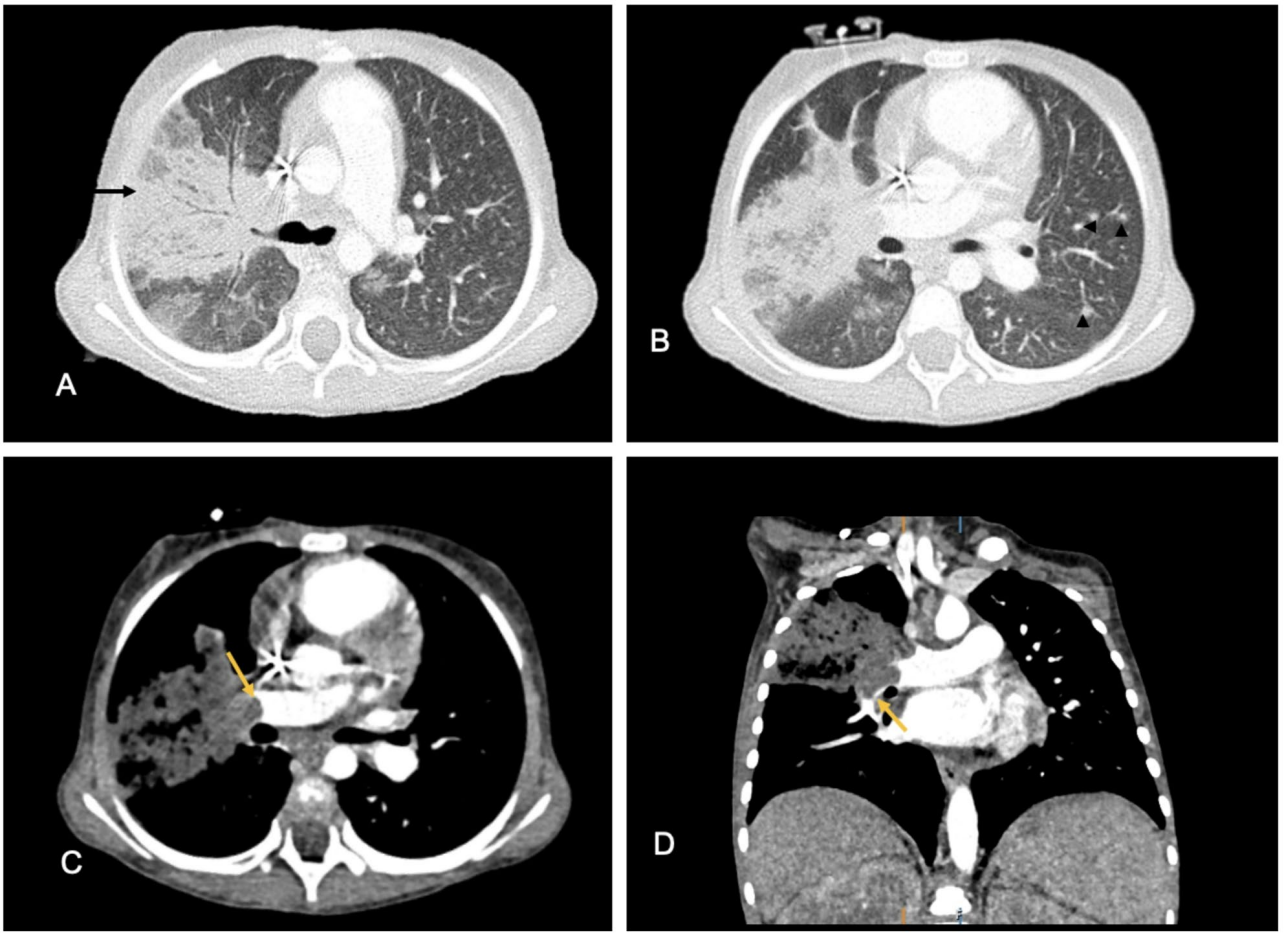
Disseminated candida infection in a 3-year-old girl with relapsed B-cell acute lymphoblastic leukemia at 10 months after CAR T-cell therapy. Imaging performed 10 months post–CAR T-cell infusion during an episode of febrile neutropenia and persistent Candida fungemia demonstrates: **A)** Axial chest CT shows patchy, multisegmented air-space consolidation with positive bronchograms in the right upper lobe, consistent with a pneumonia (arrow). **B)** Axial chest CT shows additional centrilobular nodules in the left lower love (arrowheads). **C)** Axial contrast enhanced chest CT reveals invasion of the pulmonary infiltrate into the main right pulmonary artery (arrow). **D)** Coronal contrast enhanced chest CT reveals invasion of the pulmonary infiltrate into the right lower lobe arteries (arrow)

Fungal infections have been reported weeks to months after CAR T-cell therapy, particularly in patients with prolonged neutropenia and/or extended corticosteroid therapy for CRS, ICANS or HLH. These include invasive aspergillosis, candidiasis (Fig. 12), mucormycosis, pneumocystis jirovecii and cryptococcus infections, among others. Kawata et al. reported diffuse bilateral ground-glass opacities with perihilar predominance and associated interlobular septal thickening in a “crazy-paving” pattern in a x-year old child at 4 months after CAR T-cell therapy, consistent with pneumocystis jirovecii pneumonia (PJP) [62].

Follow up imaging studies several months after CAR T-cell therapy can also reveal new, lineage-switched tumors, especially in patients treated with CAR T cells directed at B-cell antigens (CD19, CD22, and CD20). This is clinically important for radiologists, as patients with prior leukemia may present with new focal bone marrow or soft-tissue masses representing antigen-negative relapse. For example, in KMT2A-rearranged B-ALL, CD19-targeted therapy can result in the selection and proliferation of leukemic cells that had undergone myeloid or histiocytic differentiation, manifesting as myeloid or histiocytic sarcoma. Gray and Fair reported development of a myeloid sarcoma in a 23-year-old patient with ALL at 1.5 months after autologous CD19 CAR T-cell therapy, presenting as numerous FDG-avid lesions of the skin, subcutaneous tissues, muscles, osseous structures, mucosa, pleura, mediastinum, peritoneum, retroperitoneum, and testes on a PET scan [63]. An et al. reported development of a histiocytic sarcoma in a 9-year-old patient at 3 months after CD19/22 CAR T-cell therapy, presenting with massive hepatomegaly and multiple contrast-enhancing, diffusion-restricting liver lesions on an MRI scan [64].

Antigen escape can be addressed using sequential or combination CAR T-cell therapies targeting different antigens. Du and Zhang reported an 8-year-old boy with relapsed/refractory Burkitt lymphoma who received CD19-, CD22-, and CD20-directed CAR T-cell therapies [65]. After CD19 CAR T-cell there was no metabolic response, the cervical mass enlarged consistent with progressive disease by day 50. Subsequent CD22 CAR T-cell therapy caused grade 3 CRS with bilateral ground-glass opacities and pleural effusions on chest CT at day 3 that resolved by day 35. PET/CT on day 46 demonstrated disappearance of previously hypermetabolic cervical lymph nodes, but new FDG-avid submandibular nodes, indicating a transient partial remission followed by early relapse, histologically confirmed on day 63. The patient then received anti-CD20 CAR T-cell therapy, achieving gradual regression of cervical masses on ultrasound by day 28 and complete metabolic remission on whole-body PET/CT (day 64). He remained event-free for 16 months, experiencing only mild grade I CRS after CD20 CAR T-cell infusion.

### Future directions

As CAR T-cell therapy moves toward earlier treatment lines and advanced constructs - such as armored or allogeneic platforms - precise, noninvasive monitoring is becoming essential for pediatric oncology. Medical imaging must evolve to balance diagnostic accuracy with the unique need to minimize radiation and procedural burden in children. Emerging techniques like functional MRI, low-dose CT, and molecular imaging, bolstered by AI-driven analysis, hold the potential to distinguish true progression from immune-mediated inflammation and detect occult complications earlier than standard protocols. Ultimately, integrating pediatric-optimized imaging strategies with clinical biomarkers will enable a more personalized, risk-stratified approach, ensuring these transformative therapies achieve their full potential while minimizing long-term harm.

## Conclusion

CAR T-cell therapy represents a paradigm shift in treating pediatric malignancies, and radiologists play a critical role in patient management throughout the treatment timeline. Understanding the temporal sequence of expected imaging findings, from baseline tumor burden quantification through acute toxicity assessment to long-term response monitoring, is essential for accurate interpretation and optimal patient care. Radiologists must be prepared to recognize immune-mediated complications such as CRS and ICANS in the early post-infusion period, distinguish pseudoprogression from true disease progression at day 28-30, and identify atypical infections, lineage-switched relapses, and late effects during extended follow-up. As CAR T-cell therapies continue to evolve and expand to new targets and disease types, familiarity with this time-based imaging framework will enable radiologists to provide accurate assessments that directly impact clinical decision-making and patient outcomes.

## Data Availability

No datasets were generated or analyzed during the current study.
